# Human Platelet-Rich Plasma- and Extracellular Matrix-Derived Peptides Promote Impaired Cutaneous Wound Healing In Vivo

**DOI:** 10.1371/journal.pone.0032146

**Published:** 2012-02-23

**Authors:** Tatiana N. Demidova-Rice, Lindsey Wolf, Jeffry Deckenback, Michael R. Hamblin, Ira M. Herman

**Affiliations:** 1 Graduate Program in Cellular and Molecular Physiology, Sackler School of Graduate Biomedical Sciences, The Center for Innovations in Wound Healing Research, Tufts University School of Medicine, Boston, Massachusetts, United States of America; 2 Wellman Center for Photomedicine, Massachusetts General Hospital, Boston, Massachusetts, United States of America; 3 Department of Dermatology, Harvard Medical School, Boston, Massachusetts, United States of America; 4 Harvard-MIT Division of Health Sciences and Technology, Cambridge, Massachusetts, United States of America; Medical College of Georgia, United States of America

## Abstract

Previous work in our laboratory has described several pro-angiogenic short peptides derived from endothelial extracellular matrices degraded by bacterial collagenase. Here we tested whether these peptides could stimulate wound healing in vivo. Our experiments demonstrated that a peptide created as combination of fragments of tenascin X and fibrillin 1 (comb1) applied into cranial dermal wounds created in mice treated with cyclophosphamide to impair wound healing, can improve the rate of wound closure. Furthermore, we identify and characterize a novel peptide (UN3) created and modified from two naturally-occurring peptides, which are present in human platelet-rich plasma. In vitro testing of UN3 demonstrates that it causes a 50% increase in endothelial proliferation, 250% increase in angiogenic response and a tripling of epithelial cell migration in response to injury. Results of in vivo experiments where comb1 and UN3 peptides were added together to cranial wounds in cyclophosphamide-treated mice leads to improvement of wound vascularization as shown by an increase of the number of blood vessels present in the wound beds. Application of the peptides markedly promotes cellular responses to injury and essentially restores wound healing dynamics to those of normal, acute wounds in the absence of cyclophosphamide impairment. Our current work is aimed at understanding the mechanisms underlying the stimulatory effects of these peptides as well as identification of the cellular receptors mediating these effects.

## Introduction

Despite significant progress that has been achieved in our understanding of normal wound healing process and the pathologies that lead to wound chronicity, chronic wounds of differing etiology remain a significant health care burden affecting over 5 million people annually in the United States, alone [1]. In addition, acute and combat-associated wounds cause approximately 330,000 hospitalizations in this country alone [2], [3].

The importance of endogenous platelets during the early phase of the course of wound healing has been known for decades. Early on platelets accumulate at the site of injury, and participate in blood clotting and inflammatory cascades releasing interleukin 1β (IL-1β) and IL-8 necessary for monocyte adhesion and neutrophil activation respectively [4]–[6], Furthermore, activated platelets release key cellular survival factors, such as platelet derived growth factor (PDGF), vascular endothelial growth factor (VEGF) and epidermal growth factor [7], [8] which stimulate cellular migration, proliferation and angiogenesis necessary for successful wound healing. Recently [9], it has been suggested that exogenous platelets and platelet products, including platelet rich plasma extracts, might be used for stimulating wound healing as well. This work is aimed at characterization of small peptides derived from endothelial extracellular matrices and extracts of platelet rich human plasma that could be used as stimulators of cellular responses to injury. We test a hypothesis that similarly to native platelet products, platelet-rich plasma derived peptides (PDP) would stimulate cellular proliferation, migration and morphogenesis. Furthermore in this study we expanded our knowledge about another biologically active peptide isolated from endothelial extracellular matrices degraded by bacterial collagenase, which was previously identified in our laboratory [10]. PDP and extracellular matrix derived peptides (EDP) are tested in several in vitro assays and in a mouse model of impaired wound healing. Results reveal that the peptides could be used as separate entities or in combination to stimulate cellular responses to injury both in vitro and in vivo. We demonstrate increased wound re-epithelialization, granulation tissue formation and restoration of wound healing ability in animals whose healing responses had been compromised by cyclophosphamide treatment.

## Materials and Methods

### Ethics Statement

All animal protocols and experiments were approved by the Subcommittee on Research Animal Care of Massachusetts General Hospital or Institutional Animal Care and Use Committee at Tufts University and were performed in accordance with NIH guidelines.

### Cell culture

Bovine capillary endothelial cells (BCEC) were cultured as previously described [11]. Human capillary endothelial cells were grown in DMEM supplemented with 5% fetal bovine serum (Atlanta Biologicals, Inc., Lawrenceville, GA) and antibiotics (Invitrogen, Carlsbad CA).

Adult normal human epidermal keratinocytes (NHEK) were purchased from Lonza (Walkersville, MD) and grown in serum free keratinocyte growth media supplemented with human recombinant epidermal growth factor (hrEGF) and bovine pituitary extract (Invitrogen, Carlsbad CA) as per the manufacturer's instructions.

Human spontaneously transformed keratinocytes (Hacat) cells were provided by Dr. Garlick (Tufts University, Boston, MA) and cultured in DMEM supplemented with 10% fetal bovine serum (Atlanta Biologicals, Inc., Lawrenceville, GA) and antibiotics (Invitrogen, Carlsbad CA).

### Human blood platelet extracts and lysate preparation

Blood platelet extracts were prepared as follows. Pooled donor platelet rich plasma was obtained from the South Texas Blood and Tissue Center (San Antonio, TX), centrifuged at 4 C for 15–20 min at 5000 rpm. Platelet poor plasma (supernatant) was discarded and the pellet fraction was reconstituted using fresh frozen plasma, resulting in a platelet count of between 250,000 to 4,500,000 platelets per milliliter. The platelets were gently rotated to mix the contents and then snap frozen in liquid nitrogen prior to lyophilization. Multiple lots of pooled platelets were prepared in this manner and the lyophilized material from each lot was reconstituted with sterile PBS to approximately 25 mg/ml protein prior to fractionation or addition to cells as described below.

Blood bank platelet lysate was prepared from platelets acquired from Tufts Medical Center Blood Bank. Platelets were lysed using a saline solution containing 0.3% sodium citrate, 0.9% NaCl in 10 nM NaPO4, pH 6.5, centrifuged, resuspended in the same solution, centrifuged three more times, and frozen for storage.

### Gel filtration and ion-exchange chromatography [12]

Lyophilized extracts were reconstituted in sterile PBS at 25 mg/ml and applied onto a 41 cm column filled with G-150 Sephadex beads (Sigma-Aldrich, St. Louis, MO).

Samples were run at flow rate 4.5 ml/hr and collected using a fraction collector (BioRad, Hercules, CA). Fractionated samples were diluted with 0.02 M NaPO4 pH 7.2, mixed with DEAE-cellulose (Sigma-Aldrich, St. Louis, MO) for 30 minutes, and centrifuged for 1 min at 4 C in a microfuge (12 kg). The proteins were eluted as follows. After removing the supernatant, 0.05 M NaCl was added to samples, mixed for 10 min, and centrifuged. Sodium chloride solutions of different concentrations (0.10 M, 0.15 M, 0.50 M, 1.0 M) were subsequently added to DEAE-cellulose, mixed and centrifuged. The supernatants containing different protein fractions were collected, and either applied to cells after sterile dialysis vs. PBS or subjected to gel electrophoresis as below.

### Gel electrophoresis

Prior to SDS-PAGE, platelet extracts, fractions, or lysates were dialysed at 4 C to remove salts and then resuspended in sample buffer containing 2% SDS, 125 mM Tris-Cl, pH 7, 10% glycerol, 2% 2-mercaptoethanol and bromophenol blue. After electrophoresis the gels were stained overnight with 0.25% Coomassie Blue in 50% methanol, 10% acetic acid and destained in the same alcohol solution without the dye. Alternatively, the gels were silver stained with 20% silver nitrate solution and washed several times with distilled water. Developer solution containing 0.005% citric acid, 0.05% formaldehyde was then added to the gels. Once bands appeared stop solution of 5% acetic acid was added. Images of both Coomassie and silver stained gels were taken using UVP BioChemi HR camera (UVP LLC, Upland, CA).

### Western blotting

Lyophilized or solubilized and dialysed samples derived from platelet extracts, human plasma- and extracellular matrix-derived peptides were resuspended in a sample buffer and subjected to SDS-PAGE as above. Proteins were then transferred to a nitrocellulose membranes (Whatman Inc., Piscataway, NJ) and probed as previously published [13] with anti-myosin II antibody (from I. Herman, Tufts University School of Medicine; 1∶100) followed by goat anti-rabbit HRP-conjugated secondary antibody (Santa Cruz Biotechnology, Santa Cruz, CA).

### Identification of platelet-rich plasma derived peptides and ECM-derived peptides

In vitro bioassays were used to determine which fractions possessed growth promoting, migration enhancing and/or angiogenesis-inducing activities. These bioactive fractions determined by in vitro experiments were then subjected to SDS gel electrophoresis prior to Coomassie staining band excision and analysis by MS/MS mass spectrometry as previously described [10]. Results of mass spectrometric analysis revealed the presence of several protein fragments, greater than 90% of which were known and less than 5% were human derived peptides of unknown function (UN). Two of these fragments, which we named UN1 and UN2, together with a third peptide (UN3) that we created as a fusion between UN1 and UN2 joined together with a linker amino acid and synthesized at Tufts University Core Facility (TUCF).

ECM-derived peptides were identified as described previously [10] and synthesized at TUCF.

### Epithelial migration

NHEK cells (Lonza, Walkersville, MD) were used in this assay. The cells were plated in 24 well plates at a density of 1.5×10^5^ cells/well. The next day the confluent cultures were wounded using a pipette tip, washed 3 times with PBS and fed with fresh culture media. Control wells contained the basal serum free media (Lonza, Walkersville, MD). Media in the experimental wells was supplemented with 0.5 nM PDP (UN1-UN3). HB-EGF was added to media as a positive control. After the addition of the fresh media, the cells were placed onto climate-controlled microscope stage and wound closure was monitored for 4 hours. Wound sizes were measured at the time of injury and 4 h pot-injury using ImageJ software (available from NIH) and relative wound closure was then determined.

### Cell proliferation

Cell proliferation assays were performed using CEC, NHEK and Hacat cells. For the proliferation assay the cells were plated at 2×10^3^ cells per well in 3×48 well plates in basal media. One day after plating the media in control wells was replaced with media containing 1% serum for CEC or Hacat cells, or with serum free media with bovine pituitary extract and HB-EGF for NHEK. Basal media in experimental wells was supplemented with the platelet extracts (0.1–200 ng/mL) or the peptides added at 100 nM (comb1) or 250 nM (UN3). The cells were fed every other day and counted at day 5 post-plating. Three wells were used per condition and each experiment was performed in triplicate.

### In vitro endothelial morphogenesis

This assay was performed as described with minor modifications [10]. Briefly, CEC or HMDEC 7×10^4^ cells/cm^2^ were plated on growth factor reduced Matrigel at (GFR Matrigel, BD Biosciences, Bedford, MA) in DMEM supplemented with 1% BCS in the presence or absence of platelet extracts, PDP or EDP added directly to basal media or mixed with GFR Matrigel prior to gel polymerization 100 nM (comb1) or 250 nM (UN peptides). Purified recombinant basic FGF (FGF-2) or VEGF (R&D Systems, Minneapolis, MN) were used as positive controls. BCEC endothelial sprout formation was monitored at 7 h post-plating; HMDEC sprout formation was monitored at 4 h. Cell imaging was performed using an Axiovert 200 M microscope equipped with 10× objective lenses (Carl Zeiss MicroImaging, Thornwood, NY). Images were analyzed using ImageJ software (available from NIH). The total sprout length per field were measured from several experiments performed in duplicate or triplicate and quantified as described [10].

### In vivo wound healing studies

Balb/c mice were pre-treated with two doses of cyclophosphamide (CY) dissolved in sterile saline and administered by IP injection: 150 mg/kg 4 days and 100 mg/kg 1 day before wounding in order to delay wound healing as described previously [14]. Three control mice received the wounds as described below and were treated with daily application of carboxymethylcellulose (CMC), which was used in this study as a vehicle for peptide application.

Full thickness cutaneous, excisional head wounds were created in the following way. The mouse heads were depilated (Nair, Carter-Wallace, New York, NY) 15–24 h prior to wounding. For wounding procedures and during all manipulations and treatments the mice were anesthetized by an intraperitoneal injection of a ketamine-xylazine cocktail (90 mg/kg ketamine and 10 mg/kg xylazine). Full thickness excisional wounds were made with sterile 4 mm punch biopsy tool, sterile scissors and forceps. Immediately after injury wounds were dressed (Tegaderm, 3 M St. Paul, MN). The peptides were suspended in 3% CMC in PBS at concentration of 1 mg/mL (comb1) or 284 µg/mL (UN3) and injected under the dressings using 25G needle at 24 h post-injury. CMC or Regranex gels were used as negative and positive controls, respectively. All treatments were applied daily and dressings were changed as necessary.

### Tissue harvesting and staining

Collection of animal tissues was performed at 5 or 10 days post-injury. The animals were euthanized by CO_2_ inhalation and wounds, together with underlying tissues and approximately 5 mm of intact dermis surrounding the wound were then excised. The wounds then were bisected through the midline. One half of the tissue then was placed in 10% phosphate buffered formalin (Fisher Scientific, Asheville, NC), fixed for 24 h, embedded in paraffin, cut into 5 µm thick sections and stained with hematoxylin and eosin (H&E) or Trichrome stain (ThermoScientific, Asheville, NC) as per manufacturers instructions. The other half was embedded into O.C.T compound (Sakura Finetek USA, Inc., Torrance, CA 90501,) and frozen on dry ice. After freezing the tissues were cut into 5 µm thick sections and stained as described below. Imaging of Trichrome and H&E stained sections was performed with Zeiss Axiophot microscope (Carl Zeiss MicroImaging, Thornwood, NY) using 5× objective lens. The images were digitally registered and merged using Adobe Photoshop CS2 (Adobe, San Jose, CA).

### Immunohistochemical staining of frozen sections

Briefly, the sections were warmed up the room temperature for 10 min, fixed with DMEM-containing 4% paraformaldehyde and permeabilized with 0.1% Triton X-100 for 90 seconds. After blocking for 2 h with 5% goat serum, sections were incubated with rat anti-CD31 antibodies (BD Pharmingen) (1∶500) and rabbit anti-HSPG (1∶200) overnight at 4°C. Sections then were washed and co-incubated with fluorescently labeled anti-rat Alexa 546, anti-rabbit Alexa 488 secondary antibodies (Molecular Devices, Sunnyvale, CA) both at 1∶200 dilution and 16.2 µM Hoechst 33342 (Molecular Probes, Invitrogen, Carlsbad, CA) for 2 h at room temperature. After a triple wash with PBS, stained sections were mounted with PermaFluor (Lab Vision Corporation, Fremont, CA). Visualization of the slides was performed with Axiovert 200 M microscope (Carl Zeiss MicroImaging, Thornwood, NY) using 5× and 10× objective lenses.

### Analysis of wound healing and quantification of angiogenic response

Images of H&E as well as trichrome-stained sections obtained at low magnification were merged using Adobe Photoshop CS2 (Adobe, San Jose, CA). The merged images were then analyzed by two independent observers blinded to all treatment groups. To estimate the degrees of wound healing, we used a histologic scale shown in Table 1 where epithelialization and granulation tissue formation were analyzed and assigned a score in a blinded fashion.

**Table 1:** Modified histology scale for wound grading.

Score	Criteria
1	No epithelialization, no granulation tissue formed
2	No epithelialization, granulation tissue poorly formed
3	Complete epithelialization, poor granulation tissue formation
4	Complete epithelialization, well-defined granulation tissue

To quantify tissue responses to peptide treatments post injury, we assessed angiogenic activation as well as re-epithelialization. We used frozen sections stained with anti-CD31 (red) and anti-HSPG (green) antibodies as described above. The areas of antibody co-localization (yellow) representing blood vessels were quantified using ImageJ software and image co-localization tool.

### Data analysis and statistics

All in vitro experiments were performed at least triplicate. Counts obtained in cell proliferation assay, in 2D in vitro morphogenesis assay and a scratch wound model were recorded manually and analyzed using Microsoft Excel (Microsoft, Redmond, WA). Results are presented as mean ± SEM. Results of NHEK proliferation assays using fractionated platelet extracts was plotted using the ggplot2 package of R 2.10.1. Statistical significance of the in vitro findings was analyzed by two-tailed t-test. The value of p<0.05 was considered significant.

In vivo experiments were performed three times using three to five animals per each experimental group, with total number of animals of 9 to 15 per treatment. Wound healing rates determined using wound healing grades as described above and were manually recorded using Microsoft Excel (Microsoft, Redmond, WA). Data analysis was performed using one-way ANOVA test, statistical significance of the findings was then confirmed by Dunnett's and Student-Newman-Keuls tests.

## Results

### Characterization of platelet extracts lots and identification of extract-derived peptides

**Figure 1:**
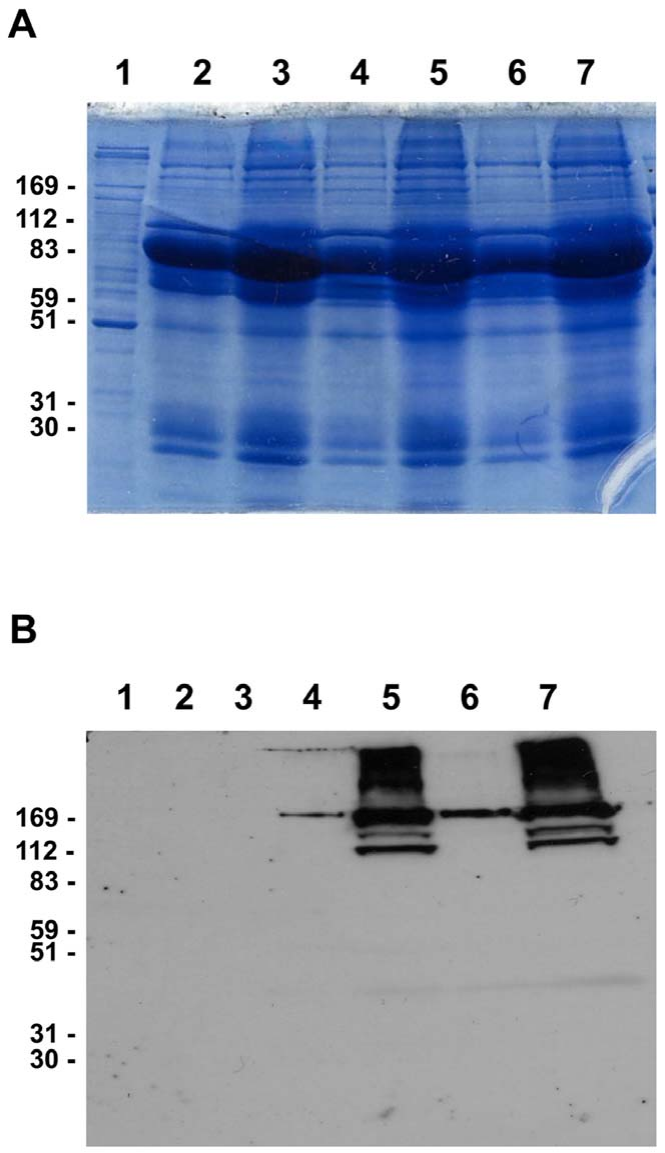
Characterization of protein composition of human platelet extracts. A – Coomassie Blue stained SDS-PAGE demonstrating the complexity of protein composition of platelet lysate and extracts. B – Western blot analysis of platelet lysate and extracts revealing the presence of myosin in lots 2 and 3, but not lot 1 extract or platelet lysate. 1- platelet lysate; 2 – platelet extract lot 1, 15 µg/mL; 3 - platelet extract lot 1, 50 µg/mL; 4 - platelet extract lot 2, 15 µg/mL; 5 - platelet extract lot 2, 50 µg/mL; 6 - platelet extract lot 3, 15 µg/mL; 7 - platelet extract lot 3, 50 µg/mL.

The platelet extracts were prepared as described in Materials and Methods and subjected to SDS-PAGE followed by Coomassie staining and mass spectrometry. Results reveal that different lots of platelet extracts contains a complex mixture of over a dozen proteins, including but not limited to albumin, immunoglobulins and cytoskeletal components, including myosin IIB (Figure 1 A-B). Interestingly, and as shown in Figure 1, some preparations were nearly free of myosin IIB, suggesting that this extract was relatively poor in platelet components when standardized for mg protein present/ml extract. In these platelet-poor preparations, plasma proteins were enriched in albumin, fibrinogen and transferrin as judged by MS/MS spectrophotometric analysis.

**Figure 2:**
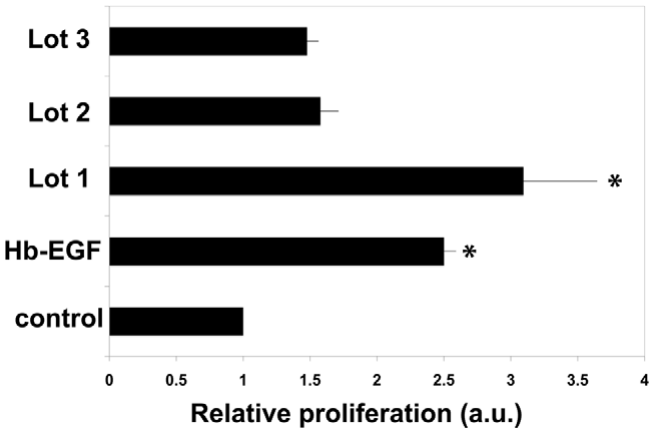
Platelet extracts stimulate epithelial proliferation in vitro. Hacat cells were plated as described in Materials and Methods in the presence or absence of at 1 µg/mL of proteins from platelet extracts (Lots 1–3). Relative proliferation compared to control is shown. Data presented as mean +/− standard error; * - indicates p<0.05.

Biological activity of the platelet extracts was then evaluated in vitro assays. As shown in Figure 2 all three lots of the extracts stimulated proliferation of transformed keratinocytes (Hacat cells). The effectiveness of the extracts preparation of different lots varied significantly. Lot 1 preparation of the extracts added to cell culture media at 1 µg/mL induced a three-fold increase in keratinocyte proliferation as compared to serum-stimulated controls. This increase of epithelial proliferation was superior to what was achieved in the presence of HB-EGF used in this study as a positive control. Lower doses of this extract preparation were less effective, inducing a 53% increased proliferation. Lots 2 and 3 of the extracts induced a 50% increase in HaCat cell proliferation while addition of 1 µg/mL extract from lots 2 and 3 have modest stimulatory effects on endothelial proliferation (Figure 2 and data not shown).

**Figure 3:**
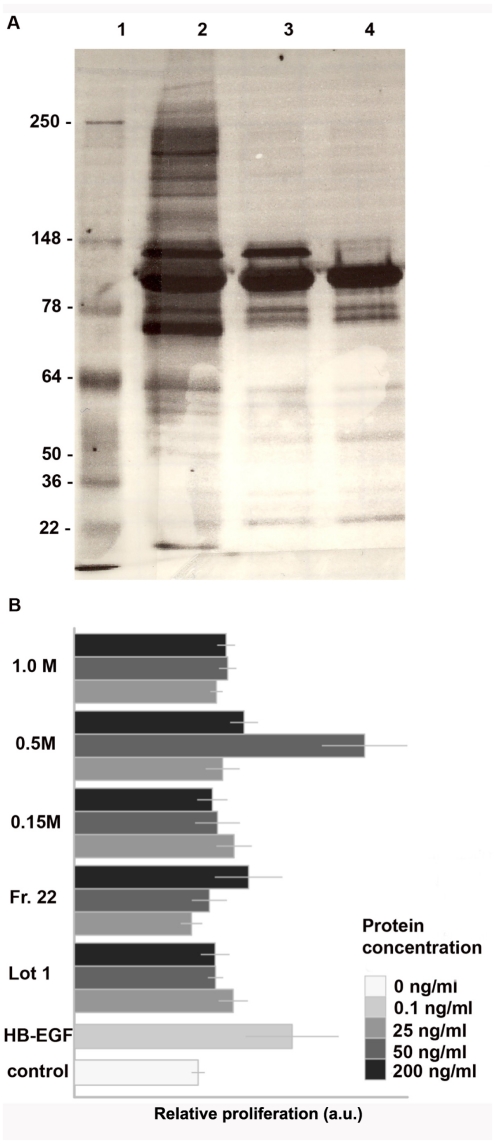
Characterization of Lot 1-derived fractions. A – silver stained SDS-PAGE demonstrating reduction of composition complexity following gel filtration and ion exchange chromatography. 1 - MW markers, 2 – unfractionated lot 1, 3- fraction 22, 4 – proteins eluted with 0.5 M NaCl. B – fractionated platelet extracts retain their biological activity toward epithelial cells. For proliferation assay NeoNHEK were plated in keratinocyte growth medium the presence or absence of unfractionated proteins from Lot 1 or fractions of Lot 1 (0.1–200 ng/mL) as indicated. HB-EGH (10 ng/mL) was used as positive control. Relative proliferation compared to control is shown. Data are presented as mean +/− standard error.

Based on these observations, we selected plasma-derived and platelet extracts that possessed pronounced biological activity and reasoned that further fractionation and enrichment of the specific cell stimulating activities might enable our identification of the biochemical entities, which promoted cell growth, migration and/or angiogenesis. To these ends, we performed gel filtration (G-150) and ion-exchange chromatography (DEAE cellulose). Gel filtration allowed for the purification of several fractions including one, fraction 22 (Figure 3A), which when applied to keratinocytes at 200 ng/ml induced a 40% increase in their proliferation rate (Figure 3 B). Further fractionation using ion-exchange chromatography and elution of the proteins with 0.5 M NaCl led to identification of significantly more potent stimulators of cellular responses. These proteins added to the cells at 50 ng/ml induced more than two-fold increase in keratinocyte proliferation (Figure 3B) and an enhancement of endothelial migration.

**Figure 4:**
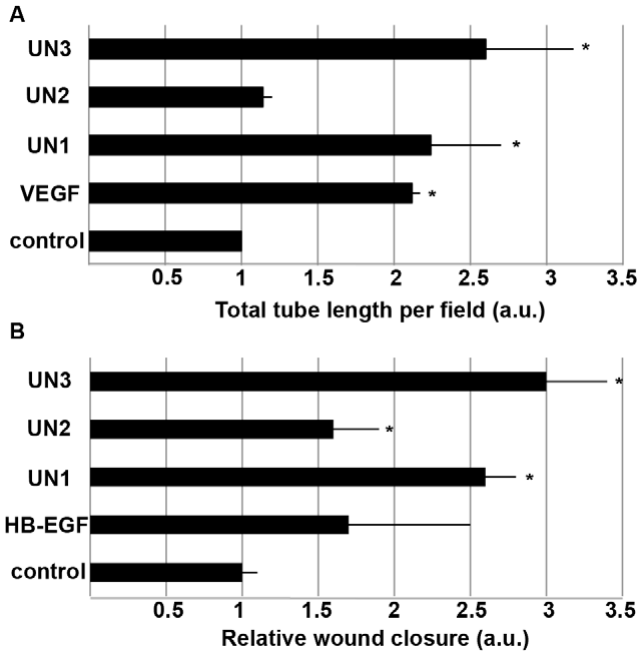
Platelet rich plasma derived peptides stimulate in vitro angiogenesis and epithelial responses to injury. A – In vitro angiogenesis assay. Human capillary endothelial cells were plated on growth factor reduced Matrigel as described in Materials and Methods. The media was supplemented with either DMEM supplemented with 1% BCS or 250 nM of UN1, UN2 or UN3 peptides. Cells that have received DMEM/1% in the presence of 10 ng/ml VEGF served as positive control. Total tube length was measured at 4 h post-plating. Relative tube length compared to control is shown. B – In vitro epithelial wound healing assay. NHEK cells were plated and injured as described in Material ad methods. The following experimental conditions were used: basal keratinocyte growth media (control), 10 ng/ml HB-EGF (positive control), 0.5 nM UN1, UN2 or UN3 peptides. Relative wound closure is shown. Data are presented as mean +/− standard error; * - indicates p<0.05.

As shown in Figure 3A, fractionation of platelet extracts significantly reduced the complexity of the protein mixtures and eliminated all high molecular weight proteins. Mass spectrometry analysis of the protein bands with molecular weight below 150 kd revealed several unnamed (UN) protein fragments, some of which possess sequence identity to fragments of human thrombin. Corresponding peptides were synthesized at TUCF. The peptides sequences are as follows: NH2-ELLESYIDGR-Amide – UN1; NH2- TATSEYQTFFNPR-Amide – UN2; NH2-ELLESYIDGRPTATSEYQTFFNPR-Amide – UN3. All three peptides stimulated angiogenesis in vitro as well as epithelial migration. However, as shown in Figure 4 a combinatorial UN3 peptide was a more potent stimulator of cellular responses than either UN1 or UN2 peptides. It induced 2.5-fold increase in a total tube length and a 3-fold increase in epithelial responses to injury (Figure 4 A and B). Therefore, we chose to use this peptide for our further in vitro and in vivo experiments.

### Identification of endothelial ECM-derived peptides

Endothelial ECM-derived peptides were isolated as described [10]. In total we have synthesized thirteen peptides. However, in this study we used the most biologically active peptide (comb1) containing fragments of both tenascin X and fibrillin 1. This peptide was chosen based on our previously published data describing its stimulatory activity toward endothelial cells in vitro [10].

### ECM and platelet extracts-derived peptides stimulate cellular responses in vitro

**Figure 5:**
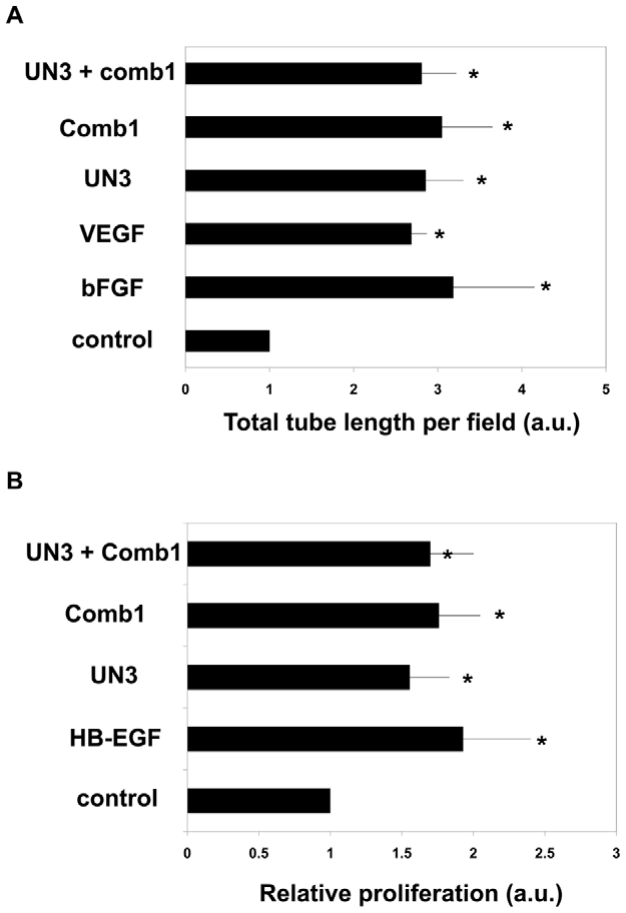
ECM-and platelet extract-derived peptides stimulate in vitro angiogenesis and epithelial proliferation. A – In vitro angiogenesis assay. Bovine capillary endothelial cells were plated on the surface of growth factor reduced Matrigel in the presence or absence of 100 nM comb1 or 250 nM UN3, or the two peptides combined. DMEM supplemented with 1% BCS was used as control, cells that have received DMEM/1% supplemented with 10 ng/ml bFGF or VEGF served as positive control. Total tube length was measured at 7 h post-plating. Relative tube length compared to control is shown. B – In vitro epithelial proliferation assay. Hacat cells were plated as described in Materials and Methods. The peptides were added at 100 or 250 nM (comb1 and UN3 respectively). Cell counting was performed at 5 days post-plating. Relative cells numbers as compared to control are shown. Data are presented as mean +/− standard error; * - indicates p<0.05.

Our previous study demonstrated that comb1 peptide had a remarkable ability to stimulate in vitro angiogenesis and increased the rates of endothelial tube formation by 2-fold [10]. In this work we wanted to determine whether a combination of the UN3 and comb1 could stimulate endothelial responses even further. This was performed using in vitro tube formation assays as described in Materials and Methods. Results reveal that the peptides used alone or in combination induce a 2–3 fold increase in the total length of tubes formed by capillary endothelial cells plated on growth factor-reduced Matrigel as compared to control cells, plated in the presence of serum (Figure 5A).

For the proliferation assays we used transformed human keratinocytes. As shown in Figure 5B, comb1 peptide significantly (75%) increased the rate of epithelial (Hacat cells) proliferation, UN3 was also effective and increased the levels of keratinocyte proliferation by 55%. A similar increase of proliferation was found for cells treated with both comb1 and UN3. Importantly, the peptides were equally effective as HB-EGF, which was used as a positive control.

### Cyclophosphamide treated mice have delayed cranial cutaneous wound healing

**Figure 6:**
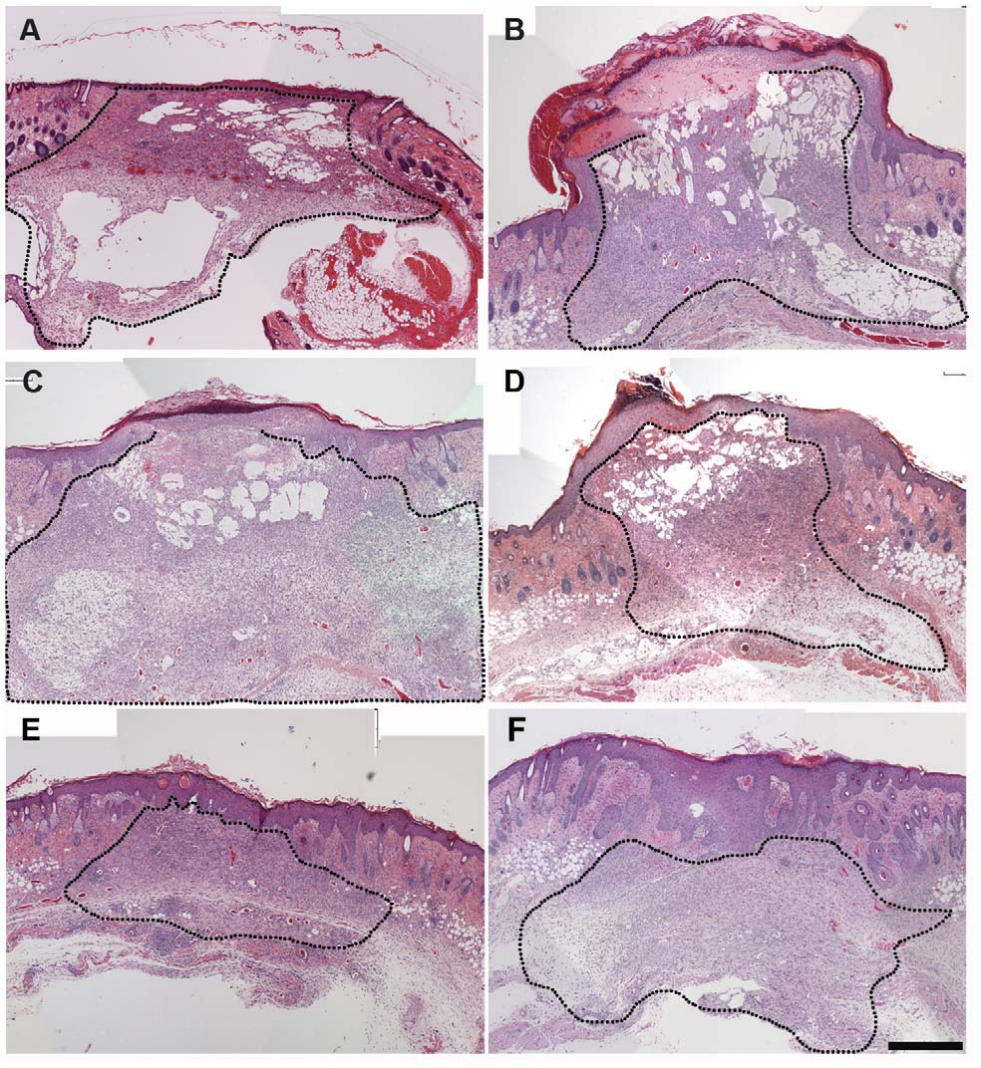
Histological evaluation of wound healing effects of the peptides in CY-treated Balb/c mice. Animals were either wounded and treated with CMC (A) or injected with CY 1 and 4 days prior to injury, wounded and treated with CMC alone (B), with Regranex (C), with UN3 (D), comb1 (E) or a combination of UN3 and comb1 (F). Wounds were excised at day 10 post-injury, formalin-fixed, sectioned and stained with haematoxylin and eosin. Dotted line delineates the wound bed. Scale bar 500 µm.

**Figure 7:**
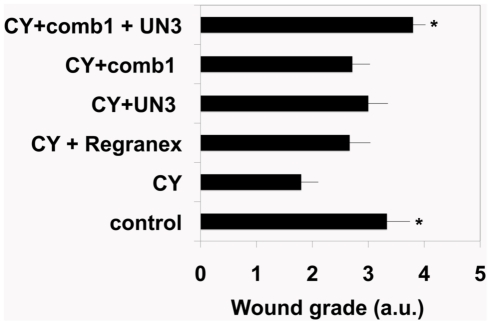
Quantitative evaluation of wound healing responses induced by the peptides in CY-treated mice. Wounds were created, treated, fixed and stained as described in Material and Methods and Figure 5. Grades were assigned to each wound based on criteria described in Table 1. At least 5 wounds were graded for each condition. Data are presented as mean +/− standard error; * - indicates p<0.05 (compared to CY+CMC treated animals).

**Figure 8:**
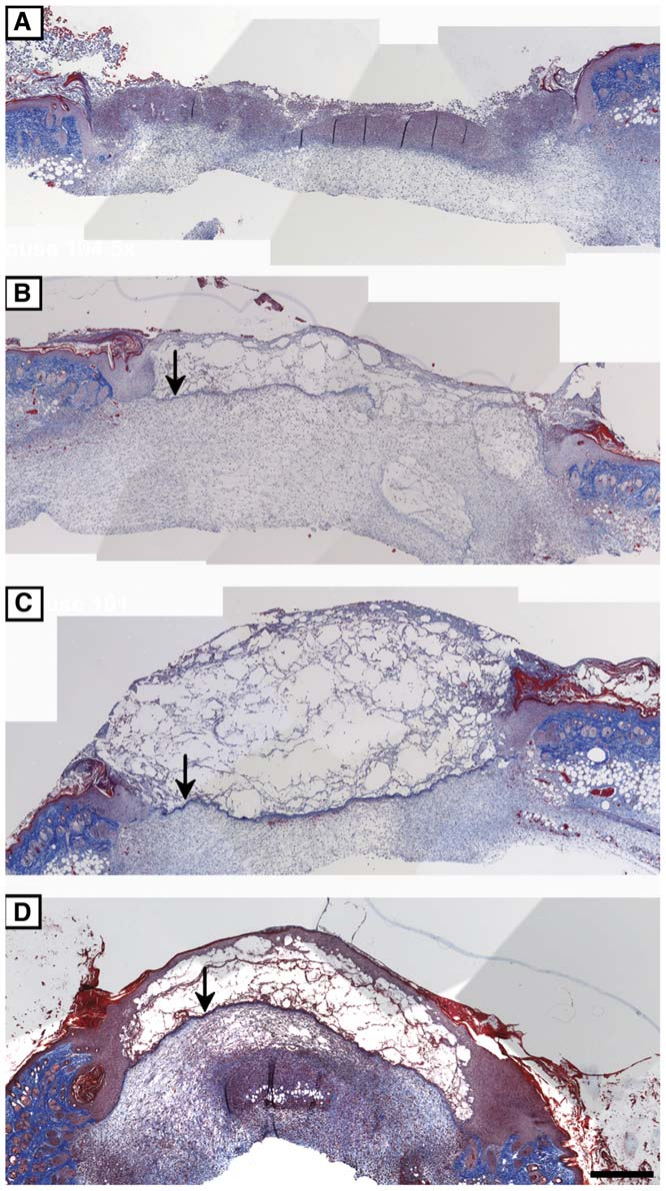
Effects of the peptides on collagen and basement membrane deposition during wound healing. Trichrome was used to stain wounds excised at day 5 post-injury. All the animals were injected with CY prior to injury. Representative sections of wounds treated with CMC alone (A), UN3 (B), comb1 (C) or a combination of UN3 and comb1 (D). Images were taken at 5× objective lens and merged using Adobe Photoshop CS2. Arrows indicate newly deposited basement membrane. Scale bar 200 µm.

In vitro studies demonstrate pro-angiogenic and pro-proliferative effects of plasma- and ECM-derived peptides, suggested that these bioactive molecules may have wound healing potential in vivo. In order to test this, we used a mouse model of impaired wound healing where Balb/c mice are pre-treated with two doses of CY at days 4 and 1 before wounding [14]. Impairment of wound healing in CY-treated mice has been observed previously [15]–[17]. However, these studies employed models of wound healing distinct from cranial dermal wounds. Therefore, first we tested whether dermal cranial wound healing would indeed be delayed in CY-treated animals. In order to estimate the degrees of healing we created and treated the wounds described in Materials and Methods, and used the grading scheme shown in Table 1 to evaluate the wound healing. Histological evaluation of the degree of wound healing revealed that CY significantly impaired cranial dermal wound healing (Figure 6A–B and Figure 7). Interestingly, this delay was more prominent at 10 days post injury and corresponded to a decrease in the number of blood vessels within the wound beds of CY treated animals compared with controls (Figures 7 and 8).

### The peptides stimulate wound healing in CY treated mice

Next we sought to evaluate whether the peptides could reverse the effects of CY and improve impaired wound healing in CY-treated Balb/c. The effects of the peptides on excisional wound healing were studied in 5 and 10 day long assays. The peptides were suspended in carboxymethylcellulose (CMC) as described above and applied into the wounds daily. CMC without the peptides was used as a control and Regranex served as a positive control.

As shown in Figures 6 and 7, single peptides as well as their combination improved wound healing in CY-treated Balb/c mice.

As evident from Trichrome stained tissue sections harvested five days after injury, the wounds in mice injected with CY alone did not display significant epithelial coverage and had only a minor accumulation of granulation tissue. At the same time wounds in peptide treated animals began to epithelialize and form granulation tissue. Collagen deposition within 5-day old wound beds was minimal in either control or peptide-treated animals.

At a later time point (10 days) there were significant differences in healing between the treatment groups. While wounds treated with CMC alone remained largely unepithelialized, wounds treated with the peptides or Regranex were covered with well-defined and stratified epithelium (Figures 6 and 7). Moreover, peptide-treated wounds were characterized by better-defined granulation tissue and by significant collagen deposition, especially in wounds treated with UN3 and a combination of UN3 and comb1 (Figure 6 E, Figure 8 D). Quantitative evaluation of H&E stained sections (Figure 7) confirmed that there were significant improvement of healing of wounds treated with the peptides as compared to controls. Interestingly, combination of the peptides (UN3+comb1) (Figures 6 and 7) further improved the outcome, suggesting the presence of a synergy between the peptides. This improvement of wound healing in the presence of the peptides was superior to that seen in Regranex-treated wounds.

### Stimulation of wound healing angiogenesis by platelet rich plasma and ECM-derived peptides

**Figure 9:**
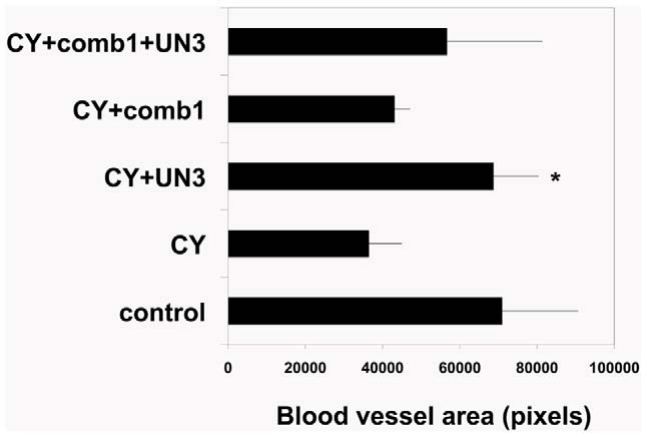
Quantitative evaluation of wound healing angiogenesis induced by the peptides. Immunohistochemistry for CD31 and HSPG was used to identify blood vessels in 10 days old wounds. The areas occupied by the blood vessels were identified by colocalized CD31 and HSPG staining and quantified using ImageJ. At least two wounds were quantified for each condition. Data are presented as mean +/− standard error; * - indicates p<0.05 compared to CY alone.

As shown previously, ECM-derived comb1 significantly improved endothelial proliferation, morphogenesis and responses to injury in vitro [10]. In addition, the data presented in this work demonstrated that plasma-derived UN3 had similar biological activity. Therefore we wanted to determine whether the wound healing stimulation by the peptides was linked to their pro-angiogenic properties. As described in Materials and Methods, in order to determine blood vessel density we performed immunostaining of frozen wound tissues with anti-CD31 and anti-HSPG antibodies. Analysis of CD31 and HSPG co-localization corresponding to blood vessels demonstrated that combined (comb1+UN3) peptides indeed stimulated wound healing angiogenesis (Figure 9). The wounds treated with the peptides had twice as many blood vessels as wounds in animals treated with CY alone (Figure 9). Importantly, the peptides reversed negative effects of CY injections on wound angiogenesis: the number of blood vessels within the wounds of CY-injected mice treated with peptides was equal to that of control (non-CY-treated) animals (Figure 9).

### Peptides promote epithelialization and basement membrane formation

Restoration of epithelial integrity is a critical step during wound healing. We assessed epithelial thickness and a degree of basement membrane formation in both control and peptide treated wounds. As shown in Figures 6 and 8 wounds treated with UN3 and comb1 combined had increased epithelial thickness at days 5 and 10 post-injury compared to CMC treated controls, and wounds treated with UN3 or comb1 separately.

Basement membrane formation was assessed using Trichrome stained histological sections of 5 days-old wounds. Results revealed the presence of well-defined basement membrane layer in peptide or Regranex treated but not in CMC treated wounds (Figure 8).

## Discussion

In our previous study we described and characterized in vitro effects of several pro-angiogenic peptides derived from endothelial ECM degraded by bacterial collagenase [10]. In this work we tested whether comb1 peptide - the most active EDP identified in vitro could stimulate cellular responses to injury in vivo. Furthermore, we have described and characterized a novel biologically active peptide derived from human platelet-rich plasma, which we termed UN3.

Platelet extracts used in this study were prepared from human donor platelet rich plasma. SDS-PAGE analysis (Figure 1) demonstrated the presence of multiple protein bands in all three extract lots studied. The complexity of extract protein composition is well known as platelet releasate have been demonstrated to contain 315 proteinaceous entities [18], including several modulators of cellular responses such as vascular endothelial growth factor, epidermal growth factor, and basic fibroblast growth factor. These proteins, as well as several less known bioactive moieties, are responsible for stimulatory effects that platelet products have on endothelial and epithelial cells which are described in the literature [19]–[21]. Similarly, our experiments using three different platelet extract preparations also demonstrated that endothelial and epithelial cell proliferation and migration can be stimulated in the presence of the extracts (Figure 2 and Figure 3). While all three lots used in this study had stimulatory effects on cells, we observed a high degree of lot-to-lot variability. For example, lot 1 preparation induced a three-fold increase of keratinocyte proliferation, while lots 2 and 3 used at identical protein concentration only moderately enhanced cellular responses by 30–50%. This variability of the biological activity of platelet products correlated with variations in their chemical compositions which were both observed in this study (Figure 1) and reported by other groups [22]. Specifically, different lots of platelet preparations vary in the abundance of components of platelet cytoskeleton (Figure 1B), cytokines and growth factors [22]. In this study we wanted to overcome lot variability of platelet extracts and identify novel biologically active moieties (peptides) possessing pro-angiogenic and wound healing properties.

The peptides were identified using the methodology similar to that used previously in our laboratory to isolate a novel beta actin-specific capping protein [12]. Specifically, we subjected platelet extracts to gel filtration and ion-exchange chromatography followed by mass spectrometry to allow for protein identification. In addition to well-known platelet extract components, we identified two novel small fragments which we named UN1 and UN2. In addition we created a combinatorial peptide UN3, which contained amino acids present in both UN1 and UN2. In vitro testing of the peptides revealed that all of them had stimulatory effects of cellular responses, however, UN3 peptide was more biologically active compared to UN1 or UN2. We also tested whether this peptide could be combined with extracellular matrix derived peptide comb1, which was identified in our previous study [10].

Our results demonstrated that both peptides use separately or in combination had stimulatory effects on cellular proliferation and morphogenesis in vitro (Figure 4A and B).

Next we sought to determine whether the peptides would retain their properties in vivo. This was tested using a mouse model of CY treated Balb/c mice that are characterized by impaired wound healing [14]. All mice used in this study received cranial dermal punch biopsies. This type of wound was used in order to minimize the wound contraction, encourage granulation tissue deposition/epithelialization and thus make mouse wound healing more resembling that of “tight skinned” animals including humans [23], [24]. Cranial wounds in CY-treated mice heal slower than in healthy counterparts (Figure 7). The peptides, especially when used in combination, significantly improved wound healing in CY-treated mice. In these animals, the peptides are more efficient than clinically used Regranex gel. Other important advantages of the peptides over PDGF and other wound healing growth factors include the relative simplicity of their synthesis and production in large quantities at relatively low cost, and their potential stability within the protease-rich wound environment due to a lack of the protease- binding sites within their structures. Furthermore, compelling pre-clinical in vitro observations point to the marked efficacy of these engineered wound healing peptides compared to peptide growth factor treatment of wounds in vitro [10].

Our in vitro experiments with endothelial cells revealed the pro-angiogenic potential of the peptides. Therefore, we tested whether similar effects could be achieved in vivo. Results revealed that the peptides could stimulate blood vessel formation within the wound bed. Similar pro-angiogenic effects were described for laminin-derived peptides [25]. These peptides induced an increase in both number and size of blood vessels at day 4 post-injury. In our experiments the effects on angiogenesis were more pronounced at a later time point – 10 days post-wounding (Figure 9). Interestingly, one of bioactive collagen IV fragments (Col4-1) identified in our previous study [10], did not enhance angiogenesis of wound healing in vivo.

Analysis of Trichrome stained sections of mouse wounds excised at day 5 post-injury showed increased epithelialization accompanied by enhanced basement membrane deposition in the presence of the peptides (Figure 8 E–H). The increase in ECM deposition could be one of the mechanisms by which the peptides stimulate wound healing. Other mechanisms are likely to include activation of ECM receptors, particularly integrins αvβ3 and α5β1 which were shown to bind to biologically active laminin-derived peptides and induce an increase in migration of both endothelial and epithelial cells [25].

Interestingly, combining the peptides before addition to the cells in vitro did not have synergistic effects (Figure 5) possibly due to direct competition between the peptides for respective receptor on the cell surface. In vivo however, the effects were more pronounced when both peptides were applied simultaneously (Figures 6 and 7). This can be linked to the fact that unlike in vivo there are more cellular receptors in relation to the peptides; therefore there is a lower chance for peptide-peptide competition.

In conclusion, we have shown that the peptide derived as a result of degradation of endothelial extracellular matrices and a peptide derived from human plasma stimulate wound healing in vivo (Figures 6 and 7). The mechanism of this stimulation is currently being investigated.
